# Hematocrit levels and thrombotic events in patients with polycythemia vera: an analysis of Veterans Health Administration data

**DOI:** 10.1007/s00277-019-03793-w

**Published:** 2019-09-24

**Authors:** Shreekant Parasuraman, Jingbo Yu, Dilan Paranagama, Sulena Shrestha, Li Wang, Onur Baser, Robyn Scherber

**Affiliations:** 1 Incyte Corporation, 1801 Augustine Cut-Off, Wilmington, DE 19803 USA; 2 STATinMED Research, 5360 Legacy Dr Ste 120, Plano, TX 75024 USA; 3 Internal Medicine – Rheumatology, University of Michigan, 3918 TC, Ann Arbor, MI 48109 USA; 4 Department of Economics, MEF University, Ayazağa St. No.4 34396, Maslak, Sarıyer, Istanbul, Turkey; 5 UT Health San Antonio MD Anderson Cancer Center, 7979 Wurzbach Rd, San Antonio, TX 78229 USA

**Keywords:** Hematocrit, Polycythemia vera, Retrospective study, Thrombosis, Thromboembolism, Thrombotic event

## Abstract

Patients with polycythemia vera (PV) have a high incidence of thrombotic events (TEs), contributing to a greater mortality risk than the general population. The relationship between hematocrit (HCT) levels and TE occurrence among patients with PV from the Veterans Health Administration (VHA) was evaluated to replicate findings of the CYTO-PV trial with a real-world patient population. This retrospective study used VHA medical record and claims data from the first claim with a PV diagnosis (index) until death, disenrollment, or end of study, collected between October 1, 2005, and September 30, 2012. Patients were aged ≥ 18 years at index, had ≥ 2 claims for PV (*ICD-9-CM* code, 238.4) ≥ 30 days apart during the identification period, continuous health plan enrollment from 12 months pre-index until end of study, and ≥ 3 HCT measurements per year during follow-up. This analysis focused on patients with no pre-index TE, and with all HCT values either < 45% or ≥ 45% during the follow-up period. The difference in TE risk between HCT groups was assessed using unadjusted Cox regression models based on time to first TE. Patients (*N* = 213) were mean (SD) age 68.9 (11.5) years, 98.6% male, and 61.5% white. TE rates for patients with HCT values < 45% versus ≥ 45% were 40.3% and 54.2%, respectively. Among patients with ≥ 1 HCT before TE, TE risk hazard ratio was 1.61 (95% CI, 1.03–2.51; *P* = 0.036). This analysis of the VHA population further supports effective monitoring and control of HCT levels < 45% to reduce TE risk in patients with PV.

## Introduction

Patients with polycythemia vera (PV) are at risk for arterial and venous thrombotic events (TEs). In a large international, retrospective study, 12% of patients with PV had an arterial TE and 9% had a venous TE over a median follow-up of 7 years [1]. Across several studies, rates of arterial and venous TEs in patients with PV ranged from 0.7 to 2.1 and 0.5 to 2.0 per 100 person-years, respectively [2–5]. Thrombotic complications have been reported as a leading cause of death for patients with PV, and prior thrombosis, particularly venous, has been identified as prognostic for overall survival [1]. Taken together, TEs may contribute to the lower survival rate observed in patients with PV compared with the general population [6].

The National Comprehensive Care Network Clinical Practice Guidelines in Oncology (NCCN Guidelines®) emphasizes prevention of thrombosis occurrence and recurrence as a primary treatment goal for PV, along with controlling disease-related symptoms [7]. This recommendation is supported by the findings of the European Collaboration on Low-Dose Aspirin in Polycythemia Vera (ECLAP) study, which observed a significant reduction in the risk of TE and death from cardiovascular causes in patients treated with aspirin [8]. In addition, hematocrit (HCT) control is associated with reduced thrombotic risk in patients with PV, as observed in the Italian Cytoreductive Therapy in Polycythemia Vera (CYTO-PV) trial [9]. In brief, the CYTO-PV trial randomized participants 1:1 to 2 groups: one with an HCT target of < 45% (low-HCT group) and the other with an HCT target of 45 to 50% (high-HCT group). Patients in the low-HCT group had a significantly lower rate of cardiovascular death and major thrombosis than those in the high-HCT group [9].

The study described here reports the results of a retrospective analysis of Veterans Health Administration (VHA) claims data. The VHA is the largest integrated healthcare system in the USA and provides real-world data on a robust patient population. The aim of this study was to investigate the relationship between HCT levels and TE occurrence among patients with PV and is the first to replicate the CYTO-PV findings in a real-world setting.

## Methods

### Database characteristics

The VHA consists of 150 medical centers and nearly 1400 community-based outpatient clinics, veteran’s centers, and domiciliary. In 2014, 9.11 million patients were enrolled in the VHA healthcare system; enrollees were primarily non-Hispanic white (82.3%) men (91.0%) under 65 years old (54.8%) [10]. VHA records provide longitudinal data, capturing the full episode of care of the VHA population. The dataset analyzed here included de-identified patient-level data from 21 Veterans Integrated Service Networks linking inpatient, outpatient, pharmacy, laboratory, enrollment, and vital sign databases.

### Study design

**Fig. 1:**
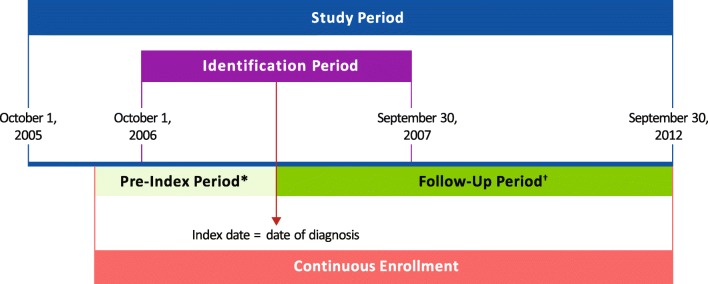
Study design schematic. * ≥ 12 months. ^†^Patients were monitored from index date until the date of the earliest event: death, disenrollment, or end of the study period

This retrospective observational claims study analyzed VHA Medical SAS® Dataset data cataloged between October 1, 2005, and September 30, 2012 (Fig. 1). Patients were monitored from the index date (defined as the date of each patient’s first claim with a PV diagnosis during the identification period [October 1, 2006, to September 30, 2007]) until death, disenrollment, or end of the follow-up period (September 30, 2012; Fig. 1).

### Patients

Patients included in the analysis had ≥ 2 claims for PV (International Classification of Diseases, Ninth Revision, Clinical Modification [ICD-9-CM] code: 238.4) ≥ 30 days apart during the identification period, were ≥ 18 years old on the index date, were continuously enrolled in a health plan with medical and pharmacy benefits from 12 months pre-index until the end of the study period, and had ≥ 3 HCT and ≥ 3 white blood cell (WBC) measurements per year during the follow-up period (WBC data analyzed and reported in a separate study). Patients with a pre-index TE were excluded from the primary analysis. This analysis included patients whose observed HCT levels were consistently within strict boundaries, always either < 45% or ≥ 45% during the follow-up period.

### Definition of TE

Patients who had a TE were defined as those with a diagnosis in medical claims during the follow-up period for at least one of the following conditions: ischemic stroke, acute myocardial infarction, deep vein thrombosis, pulmonary embolism, transient ischemic attack, peripheral arterial thrombosis, and superficial thrombophlebitis. More than one type of TE could be recorded in a single visit; therefore, the first event could include more than one type of TE per patient.

### Statistical analyses

Patient demographics and disease characteristics, baseline comorbidities, and TEs were described using descriptive statistics. Comorbid conditions were measured using the Deyo-Charlson Comorbidity Index, which assigns a weighted score ranging from 1 to 6 according to disease severity for 19 conditions [11], and the Chronic Disease Score, an aggregate comorbidity measure based on medication use (derived from pharmacy claims data) [12]. Any differences between the two HCT groups in the risk of TE were assessed using unadjusted Cox regression models based on time to first TE. For patients with a TE during follow-up, only those with ≥ 1 HCT measurement before the event were included in the Cox regression models. Time to first TE was defined as the period between index date and first claim associated with a TE diagnosis; patients were censored at the time of death, disenrollment, or end of the follow-up period. The primary analysis included patients without a history of TE before the index date; a sensitivity analysis including patients with a history of TE prior to the index period was also performed.

## Results

### Patients

**Fig. 2:**
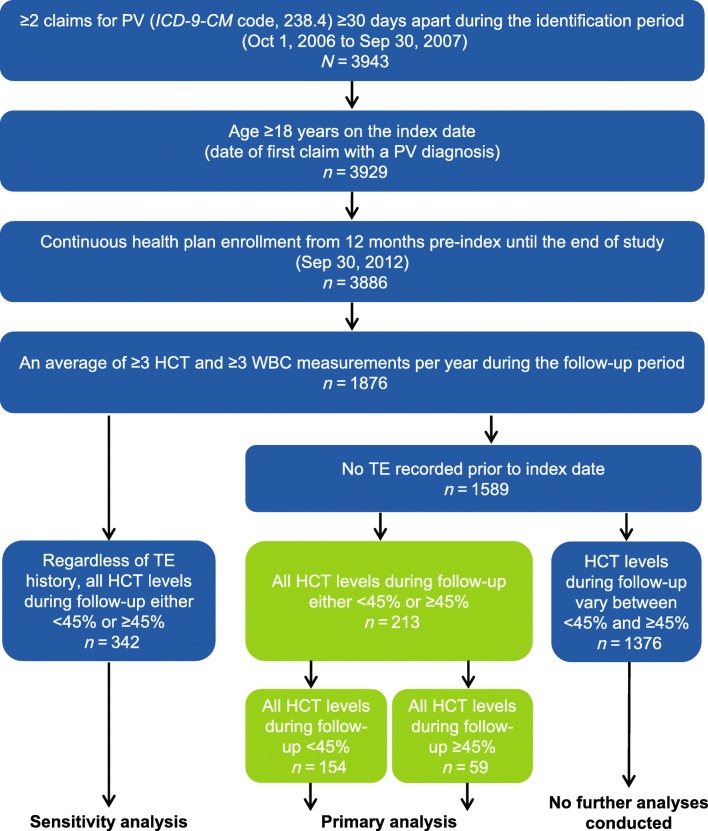
Patient selection and attrition. HCT, hematocrit; *ICD-9-CM, International Classification of Diseases, Ninth Revision, Clinical Modification*; PV, polycythemia vera; TE, thrombotic event; WBC, white blood cell

There were 3943 patients who had ≥ 2 medical claims for PV during the identification period. In total, 1876 had ≥ 3 HCT and ≥ 3 WBC measurements per year, of whom 342 had consistent HCT status throughout follow-up. Of these patients, 213 did not experience a TE during the pre-index period (HCT < 45%, *n* = 154; HCT ≥ 45%, *n* = 59; Fig. 2) and were included in the primary analysis.

Among the 213 patients included in the analysis, most were male (98.6%) and white (61.5%), with a mean (SD) age of 68.9 (11.5) years (Table 1). The mean (SD) Deyo-modified Charlson Comorbidity Index score and Chronic Disease Score were 1.46 (1.56) and 7.34 (4.37), respectively, indicating a notable pre-index comorbidity burden. The most common comorbidities were hypertension (72.8%), dyslipidemia (37.6%), chronic obstructive pulmonary disease (24.9%), and diabetes (17.4%). Use of smoking cessation therapy (used as a surrogate measure of smoking rate) was present for 23.9% of the patients.

**Table 1:** Patient demographics and clinical characteristics at index date

Characteristic	No pre-index TE and consistent HCT		
	Total (*n* = 213)	HCT < 45% (*n* = 154)	HCT ≥ 45% (*n* = 59)
Mean (SD) age, years	68.9 (11.5)	71.8 (10.7)	61.2 (10.0)
Male, *n* (%)	210 (98.6)	151 (98.1)	59 (100)
Race/ethnicity, *n* (%)			
White	131 (61.5)	97 (63.0)	34 (57.6)
Black	19 (8.9)	13 (8.4)	6 (10.2)
Hispanic	10 (4.7)	8 (5.2)	2 (3.4)
Other	53 (24.9)	36 (23.4)	17 (28.8)
Mean (SD) BMI, kg/m^2^	28.5 (12.3)	27.5 (13.6)	31.3 (6.9)
Comorbidities			
Mean (SD) Deyo-modified CCI score	1.46 (1.56)	1.64 (1.66)	0.97 (1.13)
Mean (SD) CDS	7.34 (4.37)	7.65 (4.43)	6.53 (4.10)
Common comorbidities and health-related behaviors, *n* (%)			
Hypertension	155 (72.8)	115 (74.7)	40 (67.8)
Dyslipidemia	80 (37.6)	52 (33.8)	28 (47.5)
COPD	53 (24.9)	35 (22.7)	18 (30.5)
Diabetes	37 (17.4)	29 (18.8)	8 (13.6)
Cardiovascular event	33 (15.5)	24 (15.6)	9 (15.3)
Bleeding	23 (10.8)	18 (11.7)	5 (8.5)
Asthma	6 (2.8)	5 (3.2)	1 (1.7)
Smoking^a^	51 (23.9)	28 (18.2)	23 (39.0)

*BMI*, body mass index; *CCI*, Charlson Comorbidity Index; *CDS*, Chronic Disease Score; *COPD*, chronic obstructive pulmonary disease; *HCT*, hematocrit; *TE*, thrombotic event

^a^Based on use of smoking cessation therapy in claims

### TEs during follow-up

**Fig. 3:**
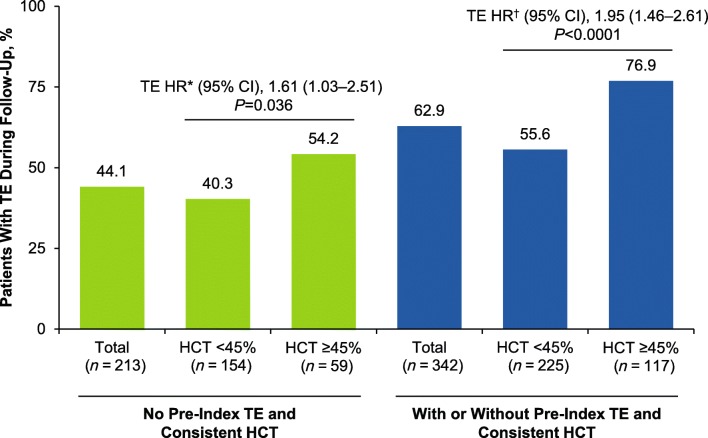
TE during follow-up. HCT, hematocrit; HR, hazard ratio; TE, thrombotic event. *HR calculation based on patients with ≥ 1 HCT value before first TE (*n* = 208). ^†^HR calculation based on patients with ≥ 1 HCT value before first TE (*n* = 322)

Among qualifying patients, 44.1% (94/213) experienced a TE during follow-up (mean follow-up, 2.3 years; rate per 100 person-years, 18.9; Fig. 3). The 3 most common types of TE recorded as the first event during follow-up were ischemic stroke (21.6%, 46/213), deep vein thrombosis (12.2%, 26/213), and transient ischemic attack (9.4%, 20/213; Table 2). Patients with HCT levels < 45% (*n* = 154; median follow-up, 2.48 years) compared with those with levels ≥ 45% (*n* = 59; median follow-up, 1.97 years) had a lower risk of TE (40.3% vs 54.2%, respectively; Fig. 3). A statistically significant increased risk of TE was observed among patients with HCT levels ≥ 45% compared with those with HCT levels < 45% (hazard ratio [HR] calculation including 208 patients with ≥ 1 HCT value before first TE, 1.61; 95% CI, 1.03–2.51; *P* = 0.036).

**Table 2:** Rates and types of TE during follow-up

	No pre-index TE and consistent HCT					
	Total (*n* = 213)		HCT < 45% (*n* = 154)		HCT ≥ 45% (*n* = 59)	
	Patients, *n* (%)	Incidence rate per 100 person-years	Patients, *n* (%)	Incidence rate per 100 person-years	Patients, *n* (%)	Incidence rate per 100 person-years
TE	94 (44.1)	18.9	62 (40.3)	16.2	32 (54.2)	27.5
Ischemic stroke	46 (21.6)	9.2	29 (18.8)	7.6	17 (28.8)	14.6
Deep vein thrombosis	26 (12.2)	5.2	21 (13.6)	5.5	5 (8.5)	4.3
Transient ischemic attack	20 (9.4)	4.0	10 (6.5)	2.6	10 (17.0)	8.6
Acute myocardial infarction	15 (7.0)	3.0	12 (7.8)	3.1	3 (5.1)	2.6
Pulmonary embolism	15 (7.0)	3.0	11 (7.1)	2.9	4 (6.8)	3.4
Peripheral arterial thrombosis	5 (2.4)	1.0	1 (0.7)	0.3	4 (6.8)	3.4
Superficial thrombophlebitis	3 (1.4)	0.6	2 (1.3)	0.5	1 (1.7)	0.9

*HCT*, hematocrit; *TE*, thrombotic event

Mean body mass index (BMI) at the index date was lower among patients with a consistent HCT < 45% who did not experience a TE compared with those who did experience a TE (26.2 vs 29.4 kg/m^2^, respectively). Patients with a consistent HCT ≥ 45% had nearly identical mean index date BMI, regardless of TE during follow-up (31.2 vs 31.3 kg/m^2^).

The sensitivity analysis conducted including patients with and without TE prior to index (*n* = 342) had similar results to the primary analysis (55.6% vs 76.9% between the < 45% and ≥ 45% groups, respectively; HR calculation including 322 patients with ≥ 1 HCT value before first TE, 1.95; 95% CI, 1.46–2.61; *P* < 0.0001).

## Discussion

This retrospective analysis of VHA claims data evaluated the relationship between HCT levels and TE occurrence among patients with PV in a real-world setting. Results from this analysis are consistent with those reported in CYTO-PV [9], which identified increased risk of TE in patients with HCT ≥ 45% versus < 45% and a risk of death from cardiovascular causes or major thrombosis that was 4 times greater in the group with high HCT. In the present study, patients with HCT levels that were consistently ≥ 45% had a 70% increased risk of TE compared with those with HCT levels that were consistently < 45%.

Patients with consistently high HCT (≥ 45%) have suboptimal control of disease and may require changes to the management of their disease, such as initiation of effective cytoreductive therapy, to improve clinical outcomes. A separate analysis of cytoreductive treatment patterns in predominantly (73.8%) high-risk patients with PV from the VHA patient population revealed that only 23.2% of patients had a record of any pharmacologic cytoreductive treatment [13], in contrast to NCCN Guidelines® [7]. Among patients with HCT levels < 45%, effective monitoring and maintenance may lead to a reduction in the risk of TE by identifying changes in HCT control and cardiovascular risk factors before the occurrence of a TE. The association between the occurrence of TE and the effectiveness of treatments used to maintain HCT values below a 45% threshold was outside the scope of this study and not assessed.

When interpreting the results of the current study, it is important to note that the VHA patient population is a unique population with key differences from the general patient population. The VHA patient population analyzed in this study was predominantly male (98.6%). Overall, rates of cardiovascular risk factors and comorbidities are higher in the VHA patient population versus the general population [14]. Baseline comorbidity scores indicated a significant comorbid disease burden, even compared with patients with PV in the general US population (Charlson Comorbidity Index score, 1.29 vs 0.8) [5]. Furthermore, several of the most common baseline comorbid conditions (i.e., hypertension, dyslipidemia, smoking [based on use of smoking cessation therapy], and diabetes) are among the risk factors typically associated with cardiovascular disease [15]. Although not recorded in this study, psychological comorbidities (e.g., posttraumatic stress disorder) [16] and psychosocial issues (e.g., homelessness) [17] have also been documented in the VHA patient population and could pose challenges in properly managing TEs. Taken together, these attributes of the VHA patient population may have contributed to the high TE rate observed in this analysis, with more than a quarter of those with no pre-index TE experiencing a TE during follow-up (rate of 18.9 per 100 person-years). In comparison, a recent observational study of patients with PV in the general US population (*N* = 8124) reported a rate of 1.43 per 100 person-years [5].

Body mass index is also an important contributor to adverse cardiovascular outcomes and risk [18]. Among patients with low HCT (< 45%), those who experienced a TE during follow-up had higher index BMI. BMI may be more indicative of TE risk than comorbidities, as scores for both Charlson Comorbidity Index and Chronic Disease Score were higher for the low-HCT (< 45%) group than the high-HCT (≥ 45%) group.

A key strength of this analysis is that the methodology used real-world data from patients in 2 groups based on consistent HCT levels < 45% or ≥ 45%, similar to the groups evaluated in the CYTO-PV study. Limitations of this study are attributable to its retrospective design and the accuracy of the VHA medical database. The sample sizes for the consistent HCT cohorts were small; however, they were similar to the population sizes recorded in the CYTO-PV study (< 45%, *n* = 182; ≥ 45%, *n* = 183) [9]. The real-world nature of the study data means that the characteristics of the two cohorts may differ, and could not be controlled for without making the model unstable. The outcome variables (e.g., TE) and other clinical conditions (e.g., PV) were identified using *ICD-9-CM* codes, which are subject to potential miscoding. This was also applicable to smoking rates, which were likely underestimated, as they were identified using *ICD-9-CM* codes for management associated with smoking cessation. Moreover, potential misclassification of HCT values could have led to information bias. Platelet counts as well as over-the-counter aspirin use were not available in the VHA database. Lastly, it is important to note that because of the large difference in sociodemographic characteristics, health status, resource use, and costs between the VHA population and the general US population, the results of this study may not be generalizable to the US population.

## Conclusion

Among VHA patients with PV, those with HCT levels consistently ≥ 45% had a significantly higher risk of TE than patients with HCT levels consistently < 45%. The results of this retrospective analysis of a real-world population are in agreement with findings from the CYTO-PV study and further support effective monitoring and management of HCT levels < 45% to reduce the risk of TE in patients with PV.

## Data Availability

The dataset supporting the conclusions in this article is available from the US Veterans Health Administration. However, restrictions apply to the availability of these data, which were used under license for the current study, and so are not publicly available.
